# Genotoype-by-sequencing of three geographically distinct populations of Olympia oysters, *Ostrea lurida*

**DOI:** 10.1038/sdata.2017.130

**Published:** 2017-09-12

**Authors:** Samuel J. White, Brent Vadopalas, Katherine Silliman, Steven B. Roberts

**Affiliations:** 1 University of Washington, School of Aquatic and Fishery Sciences, Seattle, Washington 98105, USA; 2 University of Chicago, Committee on Evolutionary Biology, Chicago, Illinois 60637, USA

**Keywords:** Genotyping and haplotyping, Population genetics, Animal breeding, Biodiversity

## Abstract

Olympia oysters are found along the west coast of North America and as the only native oyster species in the region, receive considerable attention with regard to restoration and conservation. Knowledge of genetic structure of this species is essential for resource managers. Here we provide genetic data for three distinct populations of Olympia oysters in Puget Sound, Washington, USA in the form of genotype-by-sequencing data (GBS). Specifically, this includes description of sequence data and a derived table that provides single nucleotide polymorphism (SNP) information for 10,363 loci. These data are valuable not only for resource managers responsible for restoration aquaculture practices, but can provide insight into ecological drivers of selection and diversity.

## Background & Summary

Populations of the once abundant Olympia oyster (*Ostrea lurida*), have declined precipitously along the United States west coast due to habitat deterioration, overfishing, and pollution^1,2^. These declines led the Washington Department of Fish and Wildlife to list the Olympia oyster as a Washington state ‘species of concern’. Despite reductions in wild harvest due to a lack of commercial viability, natural recovery has been limited. Recent field observations suggest that settlement and recruitment are not limiting re-establishment of the species.

The life history strategy of the Olympia oyster, by virtue of brooding larvae, may result in some degree of reproductive isolation, resulting in genetic differences among populations. Without information on genetic stock structure, inadvertent transfers of stocks for restoration aquaculture may be erasing these differences, despite well-intentioned efforts to localize broodstocks and outplants. Hatchery production typically exploits the high fecundities of marine bivalves, using few broodstock to produce large numbers of outplants^3^. Following this strategy, effective population size and genetic diversity are reduced in the very populations being ‘restored’, leaving them genetically depauperate. Thus, while census numbers of Olympia oysters in restored populations may increase, genetic diversity, and thus the resilience of the population to environmental change, may be seriously impaired. It is therefore critical to properly characterize hierarchical genetic differentiation in this iconic species.

The goal of this work was to provide essential genetic information for Olympia oysters, as there is limited genomic information for this taxa. Specifically, we provide GBS data and corresponding genotype information for populations that represent distinct geographic regions in Washington, United States. The oysters used in this study were produced in a hatchery from broodstock that represent only a subset of the population, thus it remains likely that more variation exists in the wild populations.

## Methods

### Organism sampling & nomenclature

Adult oysters were collected from three locations in Puget Sound, Washington, United States; Fidalgo Bay (N 48.478252, W 122.574845), Oyster Bay (N 47.131465, W 123.021450), Dabob Bay (N 47.850948, W 122.805694) during November and December 2012. Oysters were held for 5 months in common conditions in Port Gamble, Washington and spawned in June 2013. To ensure genetic diversity, each population from each site was allowed to spawn in 24 separate groups of 20-25 oysters. Larvae produced from each population were reared in tanks based on spawning group and settled on microcultch (very small pieces of oyster shell). Post-settlement spat were grown in four replicate screened silos and fed *ad libitum* until attaining the minimum outplant size (shell length=5 mm). In August 2013, 480 juvenile oysters (5–10 mm) from each population were placed near a site of collection, Oyster Bay (N 47.138692, W 123.017387). In November 2015, oysters from the three source populations (*n*=36) were sampled for genotyping with ctenidium tissue removed and stored −80 °C. Individuals from the Dabob Bay population were labeled 1HL_XXA, Fidalgo Bay were labeled 1NF_XXA, and Oyster Bay were labeled 1SN_XXA, where ‘XX’ is a unique identification number.

### Sample preparation and sequencing

Sample preparation, library construction, and sequencing were performed by the Beijing Genomics Institute (BGI; Beijing, China). Isolation of DNA was achieved via the salting out method. Briefly, tissue was lysed with Proteinase K, followed by ethanol precipitation of nucleic acids. Sample DNA was examined for integrity via agarose gel. An image of the gel is available in file 20160105_F15FTSUSAT0768_QC_Report.pdf (Data Citation 1). Thirty-two samples (*n*=32) from each population were selected for sequencing.

Library preparation was performed following the approach by Elshire 2011 ( ref 4). Oyster DNA and Illumina adapters containing barcodes were digested with *Ape*KI restriction enzyme (recognition site: GCWGC). Adapters were ligated to digested oyster DNA, with each individual oyster receiving a unique barcode. These libraries were pooled and subjected to polymerase chain reaction (PCR). Average insert size (219 bp) was determined via Bioanalyzer (Agilent Technologies). This pool was sequenced on a HiSeq 4000 (Illumina) as an 100 bp pair-end run.

### Bioinformatics

Beijing Genomics Institute used the Reseqtools software package (https://github.com/BGI-shenzhen/Reseqtools) to remove adapter sequences, low quality reads (reads with greater than 50% of bases with a Q value <=5), and demutliplex with the split.sh script. The script utilized the index.lst file for barcode identification and the enzyme.txt file for identifying the cut site used during library preparation (see Code Availability subsection for script files) (Data Citation 1). Demultiplexing allowed for one nucleotide mismatch in barcodes. Reads lacking barcodes were discarded.

Single nucleotide polymorphisms were identified using radsnp, a part of the NPGT software package. Loci lacking >50% of SNP data were filtered out and the remaining loci were used to generate a list of genoytpes.

### Code availability

The Reseqtools software package for demultiplexing can be found in this GitHub repository: https://github.com/BGI-shenzhen/Reseqtools. The split.sh script, the index.lst file, and the enzyme.txt file are available in an Open Science Framework (OSF) repository (Data Citation 1).

The NPGT software package used for SNP detection and genotype determination is an in-house, proprietary package used by BGI.

## Data Records

All FASTQ files corresponding to each individual oysters are available in the National Center for Biotechnology Information (NCBI) Sequence Read Archive (SRA) (Data Citation 2). The sequencing effort is encompassed by NCBI BioProject PRJNA371817 (Data Citation 3). The raw FASTQ data files (i.e., non-demultiplexed) are also available in the NCBI SRA (Data Citation 4).

Other data files described in this manuscript are stored in a publicly available OSF repository: (Data Citation 1). These data are available under a Creative Commons Attribution 4.0 International Public License, whereby anyone may freely use and adapt the data, as long as the original source is credited, the original license is linked, and any changes to the data are indicated in subsequent use.

Beijing Genomics Institute provided a quality control (QC) report of the initial samples they received, as well as an evaluation of the subsequent DNA isolations they performed: 20160105_F15FTSUSAT0768_QC_Report.pdf (Data Citation 1). The report includes agarose gel images of all samples to assess DNA integrity prior to library construction.

Demultiplexing was performed using Reseqtools and the split.sh script (Data Citation 1) provided by BGI. The split.sh script requires the enzyme.txt files (Data Citation 1) to identify the cut site and distinguish it from adapter sequences. The enzyme.txt file is a two line text file containing the *Ape*KI restriction site, formatted for use in Reseqtools. The script also requires the index.lst file (Data Citation 1) to perform demultiplexing. This is a tab-delimited text file consisting of individual sample names and their corresponding barcode.

The FastQC^5^ output files were grouped together by population and then compressed into three gzipped tarballs: oly_gbs_HL_fastqc.tar.gz, oly_gbs_NF_fastqc.tar.gz, oly_gbs_SN_fastqc.tar.gz (Data Citation 1).

A table was created to provide an ‘at-a-glance’ overview of the project in the project_seq_data_aggregation.csv file (Data Citation 1). The table consists of the following information for each individual oyster: samples name, source population, latitude/longitude of sample, protocol used (i.e., genotype-by-sequencing), sequencing platform, library type (i.e., paired-end), library insert size, number of reads generated, number of bases sequenced, file size, NCBI BioProject accession, NCBI SRA accession, NCBI BioSample accessions, NCBI SRA Experiment accessions, NCBI SRA Run accessions, and NCBI SRA Sample accessions.

An overview of sequencing results is provided in the Data.stat.csv file (Data Citation 1). This provides the number of reads sequenced (millions of bases), number of bases sequenced (megabases), GC percentage, Q20 percentage, and Q30 percentage for each sample sequenced. The Q20 and Q30 percentages are the percent of bases sequenced with Phred scores of at least Q20 (i.e., base call accuracy of 99%) and at least Q30 (i.e., base call accuracy of 99.9%), respectively^6^.

Genotype information for all individuals is contained in the Genotype.csv file (Data Citation 1). This spreadsheet provides SNP loci information for individual genotypes. Over 10,000 loci were identified (10,363) and used for uniquely genoytping individuals. Each row corresponds to an identified locus and has been assigned a unique ID. The Consensus_Seq column provides an 82 nucleotide region containing an individual SNP that is unique to at least one of the individuals. The position of this SNP within that 82 base region is indicated in the pos column. The subsequent columns represent each of the 96 individuals with the following nomenclature. The leading number and trailing letter are constants (i.e., are the same across all individuals). The first two letters indicate the population to which the individual belongs (HL—Daboby Bay; NF—Fidalgo Bay; SN—Oyster Bay). The two digit number following the underscore is a unique ID for that particular individual. An example of the nomenclature for Dabob Bay oyster #23: 1HL_23A.

Single nucleotide polymorphisms were identified in each individual. The total number of homozygous and heterozygous SNPs within each individual are documented in the SNP.stat.csv spreadsheet (Data Citation 1). Additionally, this spreadsheet provides a percent contribution of homozygous and heterygous SNPs within each individual.

## Technical Validation

Input DNA quality was evaluated by agarose gel electrophoresis and can be viewed in the QC report provided by BGI: 20160105_F15FTSUSAT0768_QC_Report.pdf (Data Citation 1). All samples used for library construction were scored by BGI as ‘Level A’, meaning the integrity of the input DNA was good and the amount of sample was sufficient for two or more library constructions.

All FASTQ sequencing data files (Data Citation 2) were evaluated using FastQC^5^ to identify any potential anomalies. Overall, the FastQC analysis results showed no presence of residual sequencing adapters and were consistent with libraries generated from DNA subject to restriction digestion: with biased ‘Per base sequence content,’ ‘Sequence Duplication Levels,’ and ‘Kmer Content’ results. This is expected in part due to *Ape*KI restriction sites present on all reads.

## Additional Information

**How to cite this article:** White, S. J. *et al.* Genotoype-by-sequencing of three geographically distinct populations of Olympia oysters, *Ostrea lurida*. *Sci. Data* 4:170130 doi: 10.1038/sdata.2017.130 (2017).

**Publisher’s note:** Springer Nature remains neutral with regard to jurisdictional claims in published maps and institutional affiliations.

## Supplementary Material



## References

[d1] Open Science Framework White S. J. Roberts S. R. 2017 http://doi.org/10.17605/OSF.IO/J8RC2

[d2] *NCBI Sequence Read Archive* White S. J. 2017 SRP099079

[d3] *NCBI BioProject* White S. J. 2017 PRJNA371817

[d4] *NCBI Sequence Read Archive* White S. J. 2017 SRR5239009

